# Supplementary material for: Diversity and Distribution of Viruses Infecting Wild and Domesticated Phaseolus spp. in the Mesoamerican Center of Domestication
Source: Viruses. 2021 Jun 16;13(6):1153. doi: 10.3390/v13061153 (PMC8235658; doi:10.3390/v13061153)
Supplement: Supplementary file 1 [file viruses-13-01153-s001.zip › Code for heatmap.pdf]

Code for heatmap

```
library(gplots)
library(RColorBrewer)
data <- read.csv("genomas_para_heatmap.csv", header=T)
rnames <- data[,1]
mat_data <- data.matrix(data[,2:ncol(data)])
rownames(mat_data) <- rnames
my_palette <- colorRampPalette(c("deepskyblue4", "white", "darkred"))(n = 299)
col_breaks = c(seq(0,96),seq(97,98),seq(99,100))
png( "heatmap genomas2.png", width = 5*300, height = 5*300, res = 300, pointsize = 6)
# row_distance = dist(mat_data, method = "euclidean")
# row_cluster = hclust(row_distance, method = "ward.D2")
col_distance=dist(mat_data,method="euclidian")
col_cluster=hclust(col_distance,method="ward.D2")
par(mar=c(0,0,2,1))
heatmap.2(mat_data,
main = "Distribution pattern",
dendrogram="column",
notecol = "black",
density.info = "none",
trace = "none",
margins = c(12,9),
col = my_palette,
keysize=1,
key.title=NA,
key.xlab="Completeness",
ColSideColors=c(rep("darkolivegreen1",10),
            rep("chocolate1",10),
            rep("chartreuse4",7),
            rep("chocolate",8),
            rep("darkgreen",9),
            rep("chocolate4",8)))
par(lend=1)
legend("topright",
      legend = c("CG", "CJ", "CN", "WG", "WJ", "WN"),
      col = c("darkolivegreen1", "chartreuse4", "darkgreen", "chocolate1",  "chocolate", "chocolate4"),
      lty=1,
      lwd=10)
dev.off()
```
