# Supplementary material for: Diversity and Distribution of Viruses Infecting Wild and Domesticated Phaseolus spp. in the Mesoamerican Center of Domestication
Source: Viruses. 2021 Jun 16;13(6):1153. doi: 10.3390/v13061153 (PMC8235658; doi:10.3390/v13061153)
Supplement: Supplementary file 1 [file viruses-13-01153-s001.zip › Supplementary Figures names and description.pdf]

viruses

Figure S1. Phylogeny of potyvirus species identified in domesticated and wild bean in the Mesoamerican center of domestication of common bean, and Nayarit, Mexico. Genome sequences of the isolates of BCMV, BCMNV, and PeMoV that were identified in this study (underlined), and some selected strains from GenBank were used for the phylogenetic analysis. The name of the isolate/strain, country of origin, date of collection, and accession numbers of the genome sequences from GenBank are shown in the taxon name. Phylogenetic trees were constructed using the Maximum-Likelihood with 1000 bootstraps and the model GTR+G+I. Nodes with less than 70% bootstrap values were collapsed.

Figure S2. Phylogeny of begomovirus species identified in domesticated and wild bean in the Mesoamerican center of domestication of common bean, and Nayarit, Mexico. Genome sequences of the isolates of BGYMV, and BLV that were identified in this study (underlined), and some selected strains from GenBank were used for the phylogenetic analysis. SiChLV (Sida chlorotic leaf virus) MerMV (Merremia mosaic virus). The name of the isolate/strain, country of origin, date of collection, and accession numbers of the genome sequences from GenBank are shown in the taxon name. Phylogenetic trees were constructed using the Maximum-Likelihood with 1000 bootstraps and the model TN93+G+I. Nodes with less than 70% bootstrap values were collapsed.

Figure S3. Phylogeny of alphaendornavirus species identified in domesticated and wild bean in the Mesoamerican center of domestication of common bean, and Nayarit, Mexico. Genome sequences of the isolates of PvEV-1, and PvEV-2, that were identified in this study (underlined), and some selected strains from GenBank were used for the phylogenetic analysis. The name of the isolate/strain, country of origin, date of collection, and accession numbers of the genome sequences from GenBank are shown in the taxon name. Phylogenetic trees were constructed using the Maximum-Likelihood with 1000 bootstraps and the model GTR+I. Nodes with less than 70% bootstrap values were collapsed.

Figure S4. Phylogeny of isolates of CPMMV identified in domesticated and wild bean in the Mesoamerican center of domestication of common bean, and Nayarit, Mexico. Genome sequences of the isolates of CPMMV that were identified in this study (underlined) and some selected strains from GenBank were used for the phylogenetic analysis. The name of the isolate/strain, country of origin, date of collection, and accession numbers of the genome sequences from GenBank are shown in the taxon name. Phylogenetic trees were constructed using the Maximum-Likelihood with 1000 bootstraps and the model GTR+G+I. Nodes with less than 70% bootstrap values were collapsed.

Figure S5. Genome organization and phylogeny of bean-associated cytorhabdovirus isolate CN3 (BaCV-CN3). (A) Genome structure of BaCV-CN3 showing the seven ORF and the putative genes that codified (N, P, P3, P4, M, G, and L), as well as the position of the putative transcription initiation sequence (TIS) and the intergenic conserve consensus sequence (ICCS). (B) Conserved motif sequences in the intergenic regions and consensus sequence. The sequence in italic corresponds to the P3 gene start. (C) Phylogenetic tree of cytorhabdovirus RdRp amino acid sequences (L protein) from 21 cytorhabdoviruses constructed using the Maximum-Likelihood (LG+G+I+F model) with 1000 bootstraps. BaCV-CN3 is underlined.
