# Supplementary figures and images for: Diversity and Distribution of Viruses Infecting Wild and Domesticated Phaseolus spp. in the Mesoamerican Center of Domestication
Source: Viruses. 2021 Jun 16;13(6):1153. doi: 10.3390/v13061153 (PMC8235658; doi:10.3390/v13061153)

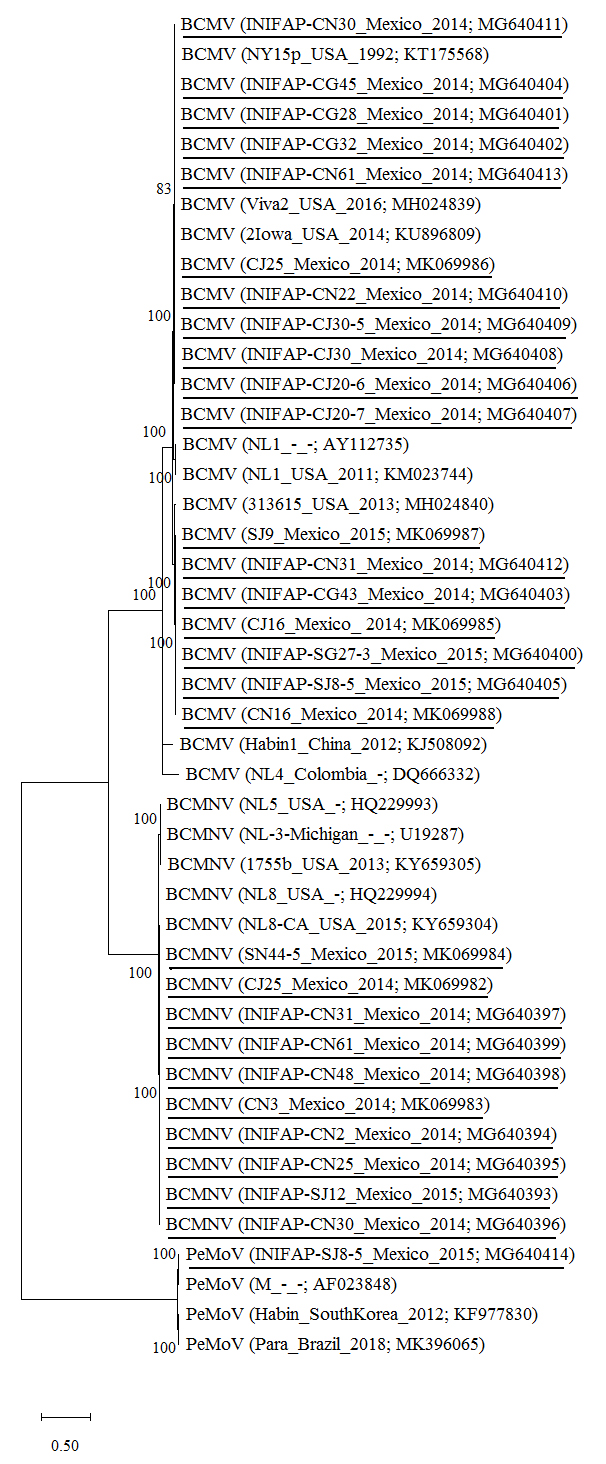

Supplement: Supplementary file 1 [file viruses-13-01153-s001.zip › Figure S1.jpg]

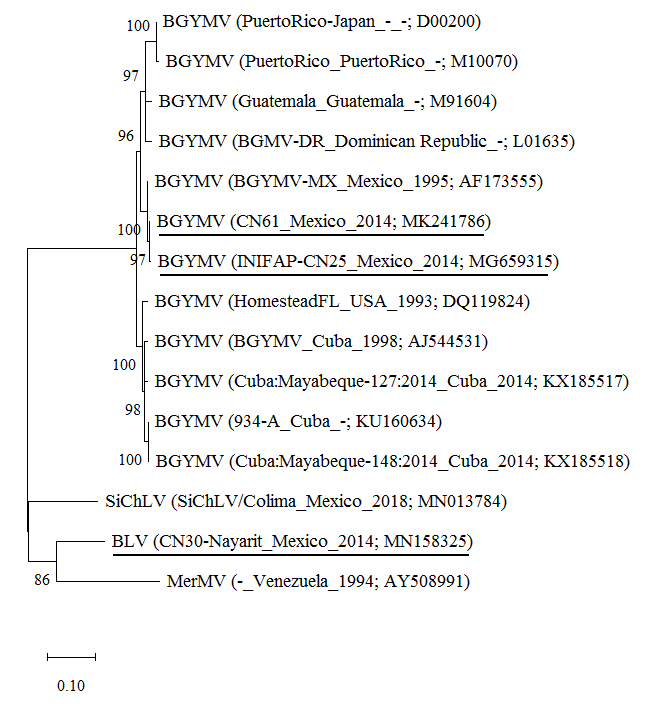

Supplement: Supplementary file 1 [file viruses-13-01153-s001.zip › Figure S2.jpg]

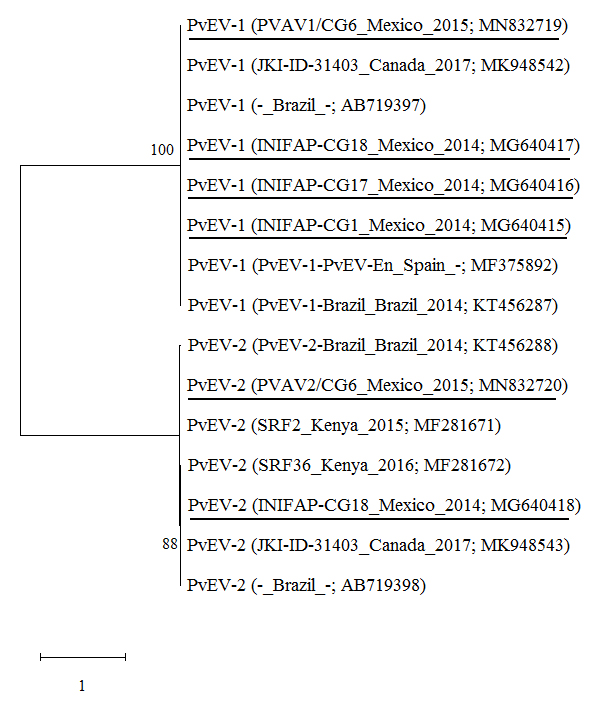

Supplement: Supplementary file 1 [file viruses-13-01153-s001.zip › Figure S3.jpg]

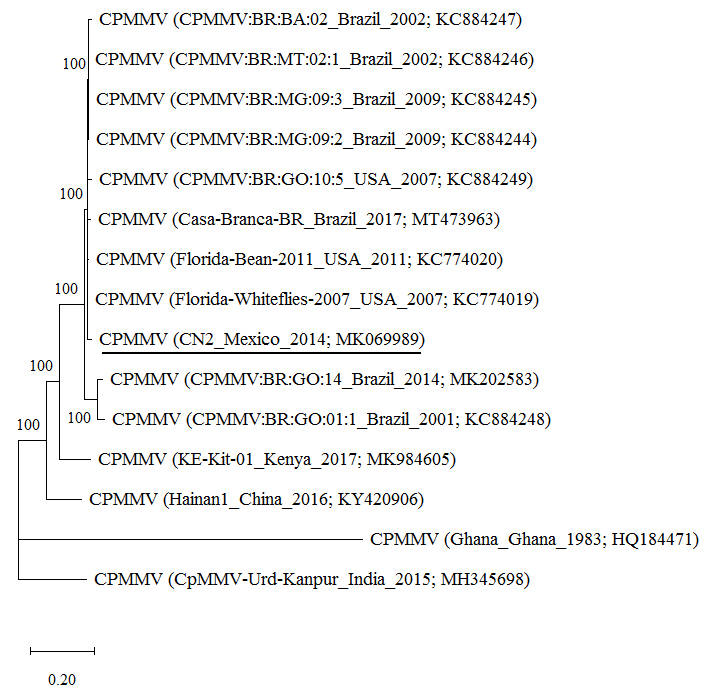

Supplement: Supplementary file 1 [file viruses-13-01153-s001.zip › Figure S4.jpg]

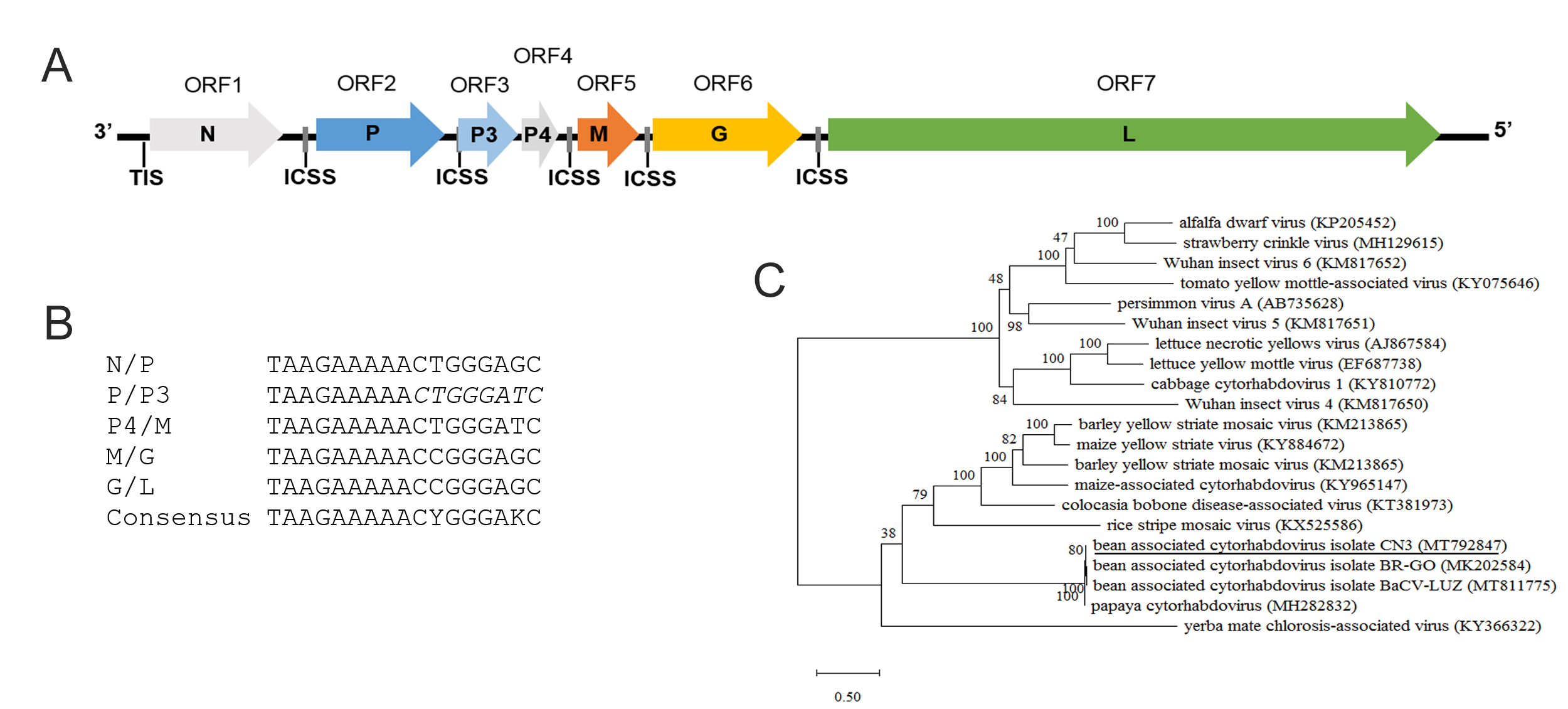

Supplement: Supplementary file 1 [file viruses-13-01153-s001.zip › Figure S5.jpg]
